# Transcriptomics reveals the molecular mechanisms of flesh colour differences in eggplant (*Solanum melongena*)

**DOI:** 10.1186/s12870-022-04002-z

**Published:** 2023-01-04

**Authors:** Tao Tao, Wei Hu, Yang Yang, Min Zou, Shanshan Zhou, Shibing Tian, Yongqing Wang

**Affiliations:** Vegetable and Flower Institute of Chongqing Academy of Agricultural Sciences, Chongqing, 401329 China

**Keywords:** Chlorophyll, Chloroplast, Eggplant (*Solanum melongena L.)*, Flesh colour, Transcriptome

## Abstract

**Background:**

Fruit flesh colour is not only an important commodity attribute of eggplant but is also closely related to maturity. However, very little is known about its formation mechanism in eggplant.

**Results:**

Two inbred lines of eggplant, green 'NC7' and white 'BL', were used in this study to explain the differences in flesh colour. Transcriptome sequencing results revealed a total of 3304 differentially expressed genes (DEGs) in NC7 vs. BL. Of the DEGs obtained, 2050 were higher and 1254 were lower in BL. These DEGs were annotated to 126 pathways, where porphyrin and chlorophyll metabolism, flavonoid biosynthesis, and photosynthesis-antenna proteins play vital roles in the colour formation of eggplant flesh. At the same time, Gene Ontology (GO) enrichment significance analysis showed that a large number of unigenes involved in the formation of chloroplast structure were lower in BL, which indicated that the formation of chloroplasts in white-fleshed eggplant was blocked. This was confirmed by transmission electron microscopy (TEM), which found only leucoplasts but no chloroplasts in the flesh cells of white-fleshed eggplant. Several genes encoding ERF and bHLH transcription factors were predicted to participate in the regulation of chlorophyll biosynthetic genes.

**Conclusions:**

The results of this study indicated that differences in the gene expression of the chlorophyll metabolic pathway were the main cause of the different flesh colour formations. These findings will increase our understanding of the genetic basis in eggplant flesh colors formation mechanism.

**Supplementary Information:**

The online version contains supplementary material available at 10.1186/s12870-022-04002-z.

## Introduction

Eggplant *(Solanum melongena L.)* is the fifth most economically important vegetable crop in the Solanaceae family and is mainly produced in China and India [1]. In China, eggplant fruit has evolved into 11 types, such as round ball, oval, long tube, long strip and short horn. Fruit colour has also evolved into white, green, purple, black purple and 8 other colours [2]. Fruit colour is one of the most intuitive qualities of horticultural crops and can be used as not only an evaluation standard of fruit maturity but also an important factor affecting consumers' purchase choices [3]. At the same time, markets in different regions have diverse needs for the colour of eggplant fruit, and the difference in colour will even directly affect the commodity value of eggplant. Therefore, research on the mechanism of fruit colour formation is very important for eggplant breeding.

The pigments of plants were mainly divided into four categories according to the chemical structure of metabolites, including pyrrole derivatives, polyene pigments, phenolic pigments and ketoquinone derivative pigments. The colour of eggplant peel is mainly determined by pyrrole derivatives and phenolic pigments [4]. However, unlike the peel, the main reason for the difference in flesh colour of eggplant is the presence or absence of chlorophyll or the degree of chlorophyll accumulation or degradation during fruit development. Wild species of Solanum crops usually have chlorophylls and carotenoids in the fruit flesh, which result in a less white flesh [5]. The chlorophyll metabolism pathway has been studied in petals in many species, such as carnation [6], chrysanthemum [7] and petunia [8]. Low expression of *Lhcb1*, *Lhcb3* and *POR* in chlorophyll synthesis leads to the chlorosis of tea leaves [9]. 5-Aminolevulinic acid (ALA) biosynthesis is the rate-limiting step of chlorophyll synthesis, and ALA is formed from glutamyl-tRNA by two enzymatic steps. The *HEMA* encodes the glutamyl-tRNA reductase (GluTR), the first ALA synthesis enzyme [10].

At present, studies on the colour difference of eggplant mainly focus on the peel and less on the flesh [11–13]. However, the flesh colour of eggplant is also important, as it not only affects the fruit ripening process but is also related to a variety of physiological active substances, such as rutin and chlorogenic acid, related to human health [1, 3, 14–16]. Anthocyanin and chlorophyll are the two main pigments in fruit colouration of plants and have been well studied in many plants. However, the mechanisms of pigment formation between different species have their own characteristics. Competition between metabolic pathways, mutations in structural genes or regulatory genes, and the activity of microRNAs will all affect the formation of fruit colour. Therefore, the study of eggplant flesh colour can reveal its specific genes and regulatory mechanisms, bringing new knowledge to this field. In this study, using eggplant with green and white flesh colour as research materials, the RNA-seq method was used to compare transcriptome profiles to identify key metabolic pathways and genes involved in eggplant flesh colour. Our work will provide relevant information for understanding the genetic basis of eggplant fruit apparent traits and the developmental potential of healthy eggplant.

## Methods

### Plant materials

**Fig. 1:**
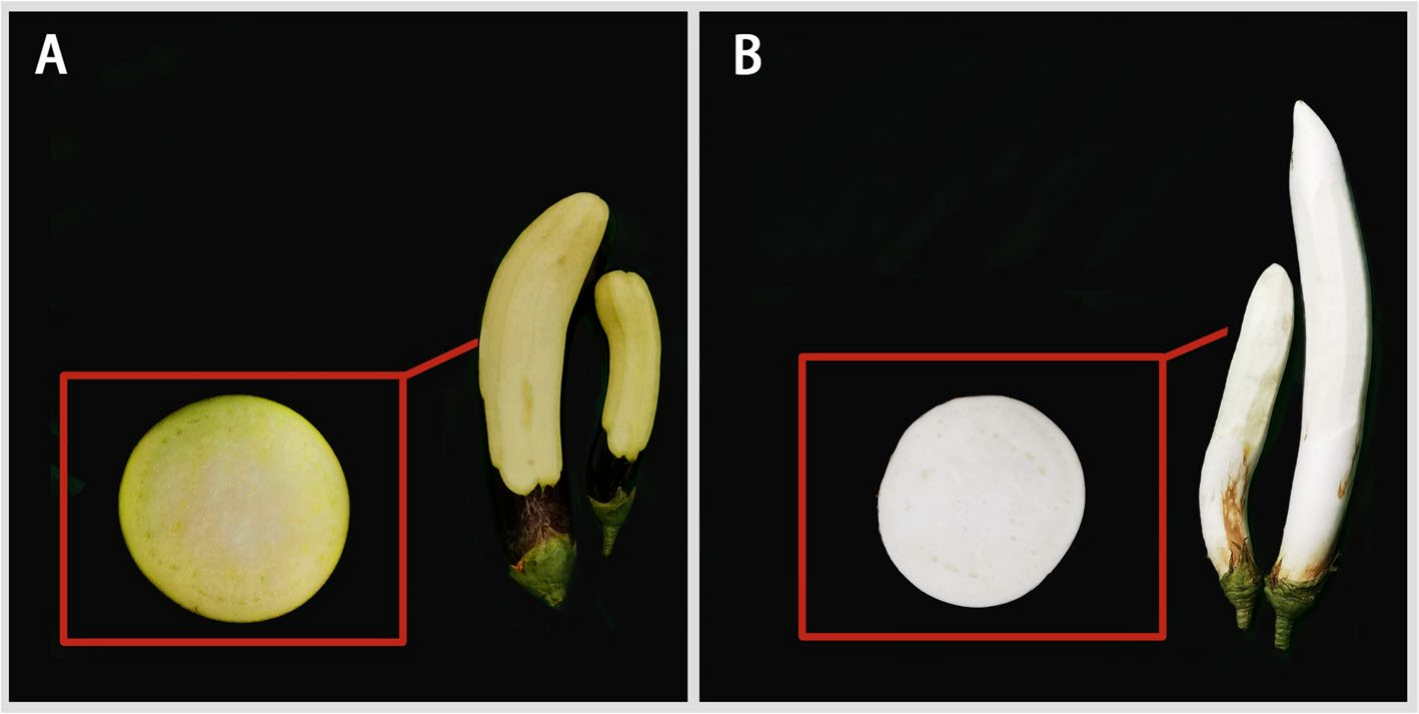
Phenotypes of (**A**) the green-fleshed eggplant cultivar NC7 and (**B**) the white-fleshed eggplant cultivar BaiLong (BL)

Two genotypes of eggplant (*Solanum melongena L.*) with different flesh colours were used in this study (Fig. 1). BL is a white-fleshed eggplant that is an inbred line of the commercial variety "Bailong" cultivated by the vegetable research institute of the Guangdong Academy of Agricultural Sciences. NC7 is a green-fleshed eggplant that is an inbred line of the commercial variety "Britta". The experiments were conducted on the Chongqing branch of the National Vegetable Improvement Center (29°21′ N, 106°18′ E), Chongqing, China. A random block design was adopted, with row spacing of 0.8 m and a plant spacing of 0.5 m. All plants adopted a unified cultivation management method and fertilization measures. Samples were collected when the eggplant fruit grew to commercial value and were stored at − 80 °C.

### RNA extraction, library preparation, and sequencing

Total RNA was extracted from the flesh of each treatment with TRIzol reagent (Invitrogen, Thermo Fisher, MA, USA) according to the manufacturer’s instructions. RNA contamination and RNA integrity number (RIN) were monitored on 1% agarose gels and an Agilent 2100 Bioanalyzer system (Agilent Technologies, CA, USA), respectively. According to previous methods [17], cDNA libraries were constructed using 500 ng of total RNA per sample with single-end 60-bp reads. Libraries were then sequenced on six lanes of a HiSeq 2500 System (Illumina) using the SR60 protocol. The output was 3 million reads per sample. Fastp v 0.19.3 was used to filter the original data, mainly to remove reads with adapters. When the N content in any sequencing reads exceeded 10% of the base number of the reads, the paired reads were removed. When the number of low-quality (Q <  = 20) bases contained in reads exceeded 50% of the bases of the reads, these paired reads were removed. High-quality clean data were applied to the downstream analyses.

### RNA-seq data analysis

Reference genome and gene model annotation files were downloaded from http://eggplant-hq.cn/Eggplant/home/index [18]. The index of the reference genome was built, and paired-end clean reads were aligned to the reference genome using HISAT v2.1.0 [19]. FeatureCounts v1.6.2 [20] was used to calculate the gene alignment and the fragments per kilobase of exon model per million mapped fragments (FPKM) of each gene based on the gene length. DESeq2 v1.22.1 was used to analyse the differential expression between the two groups, and the *P* values were corrected using the Benjamini & Hochberg method. Corrected *P* value and |log2foldchange| were used as the thresholds for significant differential expression. The threshold *p* value in multiple tests to judge the significance of gene expression differences was based on the false discovery rate (FDR) method. The screening conditions for differential genes were |log2Fold Change|> = 1 and FDR < 0.05 [21].

The GOseq R package was used to analyse Gene Ontology (GO) enrichment with DEGs [22]. The adjusted *P* value of significantly substantiated GO terms was less than 0.05. The KEGG pathways enriched with DEGs (FDR < 0.05) were detected using KOBAS 2.0 software based on the method of overrepresentation analysis (ORA) [23–26].

### RNA-seq data validation by qRT–PCR

RNA-seq samples (in triplicate) were used for quantitative real-time PCR (qRT–PCR) to test the dependability of the transcriptome results following descriptions of our previous method [27]. Total RNA (1 mg) was used to synthesize first-strand complementary DNA in combination with oligo (dT)-18 as a primer and M-MuLV reverse transcriptase (TaKaRa, Kusatsu, Japan). qRT–PCR (ABI 7900HT; Applied Biosystems, CA, USA) was performed in a 20-µL reaction system using a LightCycler 480 SYBR Green I Master kit according to the manufacturer’s protocols. Each sample analysis was repeated at least three times. Primer Premier 5.0 (Premier Biosoft, CA, USA) software was used to design specific primers, and all primers are summarized in Table S3. The PCR products were quantified by the 2^−ΔΔCt^ method with normalization to the level of *GAPDH*(*GAPDH*;Smechr0300714) [28]. We estimated the Pearson correlation coefficient between the gene expression profiles in the qRT–PCR and RNA-seq data in R version 3.6.3.

### Transmission electron microscope (TEM) sample preparation and observation

The samples were horizontally cut, and three square samples (0.5 cm × 0.5 cm) were taken from the centre to the edge of the cross section. After soaking with 2.5% glutaraldehyde for 4 h, the cells were fixed with osmic acid at 4 °C for 4 h and then dehydrated with ethanol. Once embedded in Spurr’s resin at 70 °C for 8 h, thin sections were cut from the samples with an LKB-V ultramicrotome (LKB, Sweden) and placed on 250-mesh grids [29]. Afterwards, a transmission electron microscope (Hitachi HT-7700, Tokyo, Japan) was used to observe and take pictures.

### Determination of chlorophyll content

Chop and mix the fresh eggplant pulp tissue, and then mash it into homogenate. Weigh 2 g sample to determine chlorophyll content. Chlorophyll contents were assessed using a spectrophotometer at 663 and 645 nm after soaking the samples in 15 mL of 95% ethanol for 48 h in darkness. The calculations used the methods described in previous research [30], and the contents are expressed in ${\mathrm{mg g }}^{-1}$ (FM).

$$\mathrm{Chlorophyll}\;\mathrm a\;(\mathrm{mg}\mathrm L^{-1})={12.72\mathrm A}_{663}-{2.59\mathrm A}_{645}$$

$$\mathrm{Chlorophyll}\;\mathrm b\;(\mathrm{mg}\mathrm L^{-1})={22.88\mathrm A}_{645}-{4.67\mathrm A}_{663}$$

The concentration of chlorophyll (a + b) = chlorophyll a + chlorophyll b = 8.05 *A*_663_-20.29 *A*_645_.

Calculation of chlorophyll contents in fruit flesh:

$$\mathrm{X}=0.25\mathrm{C}/\mathrm{W}$$

X- Chlorophyll content.

C- Concentration of chlorophyll in the extract.

W- FM of the sample.

### Statistical analysis

The data in the tables and figures are expressed as the means of all replicates ± SD. Each treatment had three biological replicates. Data were statistically analysed by analysis of variance using statistical software (SAS version 9.2; SAS Institute, Cary, NC) [31]. Statistical significance was assessed using the least-significant difference (LSD) test (*P* < 0.05). All figures were drawn using Origin2019 Pro.

## Results

### Sequencing data statistics

RNA-seq was performed on the flesh of NC7 and BL. A total of 40.9 Gb of clean reads were obtained. The Q30 values of the 6 samples were 93.95–95%, with an average of 94.61%, indicating that the sequencing data were of high quality and reliability and that the data could be used for subsequent analysis (Table S1). A total of 3304 differentially expressed genes (DEGs) were obtained and annotated from NC7 vs. BL (Table S2).

### Analysis of DEGs between green and white-coloured flesh in eggplant

**Fig. 2:**
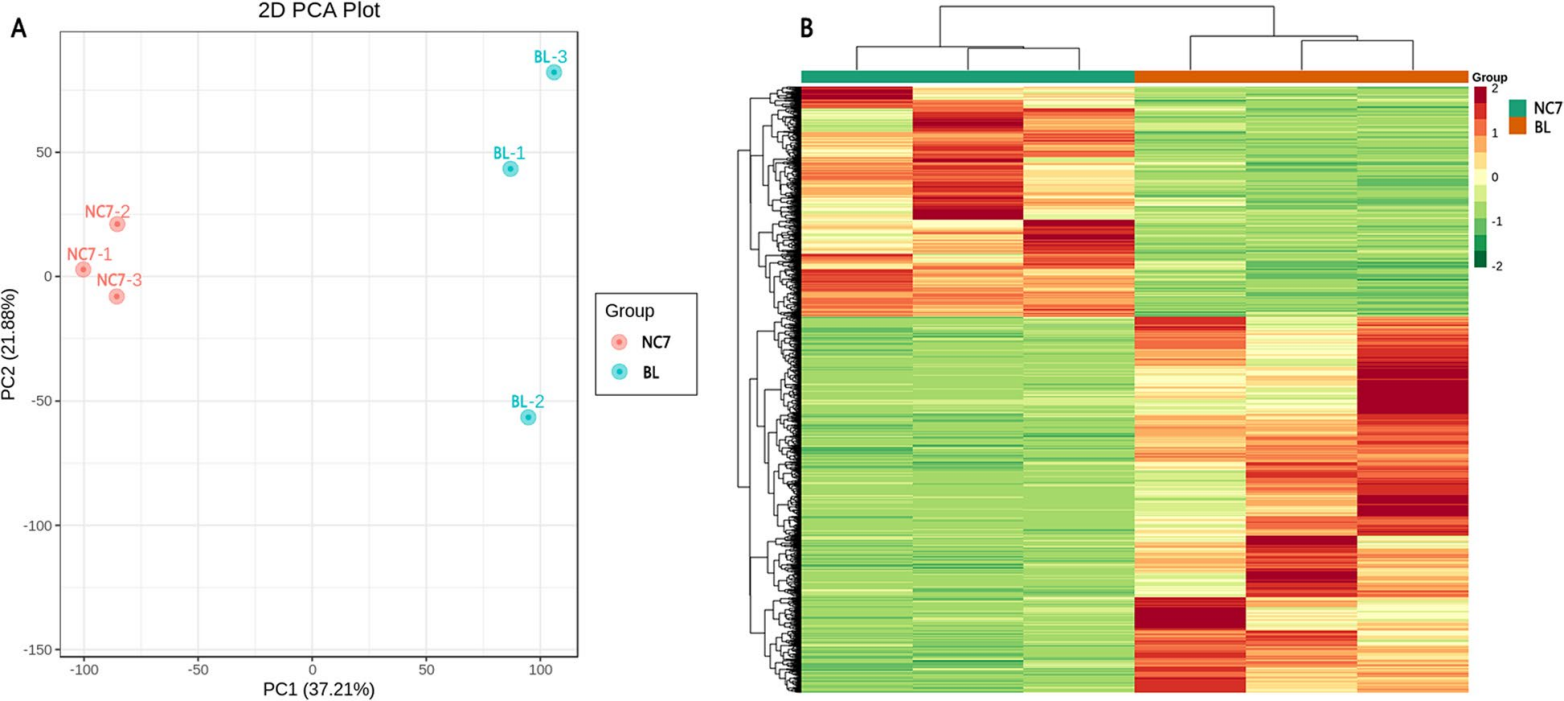
Gene expression analysis of eggplants with green and white flesh. **A** Principal component analysis (PCA) based on FPKM data. **B** Clustering analysis of DEGs between the green- and white-fleshed samples. The colour scale from green to red in the heatmap represents the normalized FPKM value using the Row Z score. NC7: green-fleshed eggplant; BL: white-fleshed eggplant

**Fig. 3:**
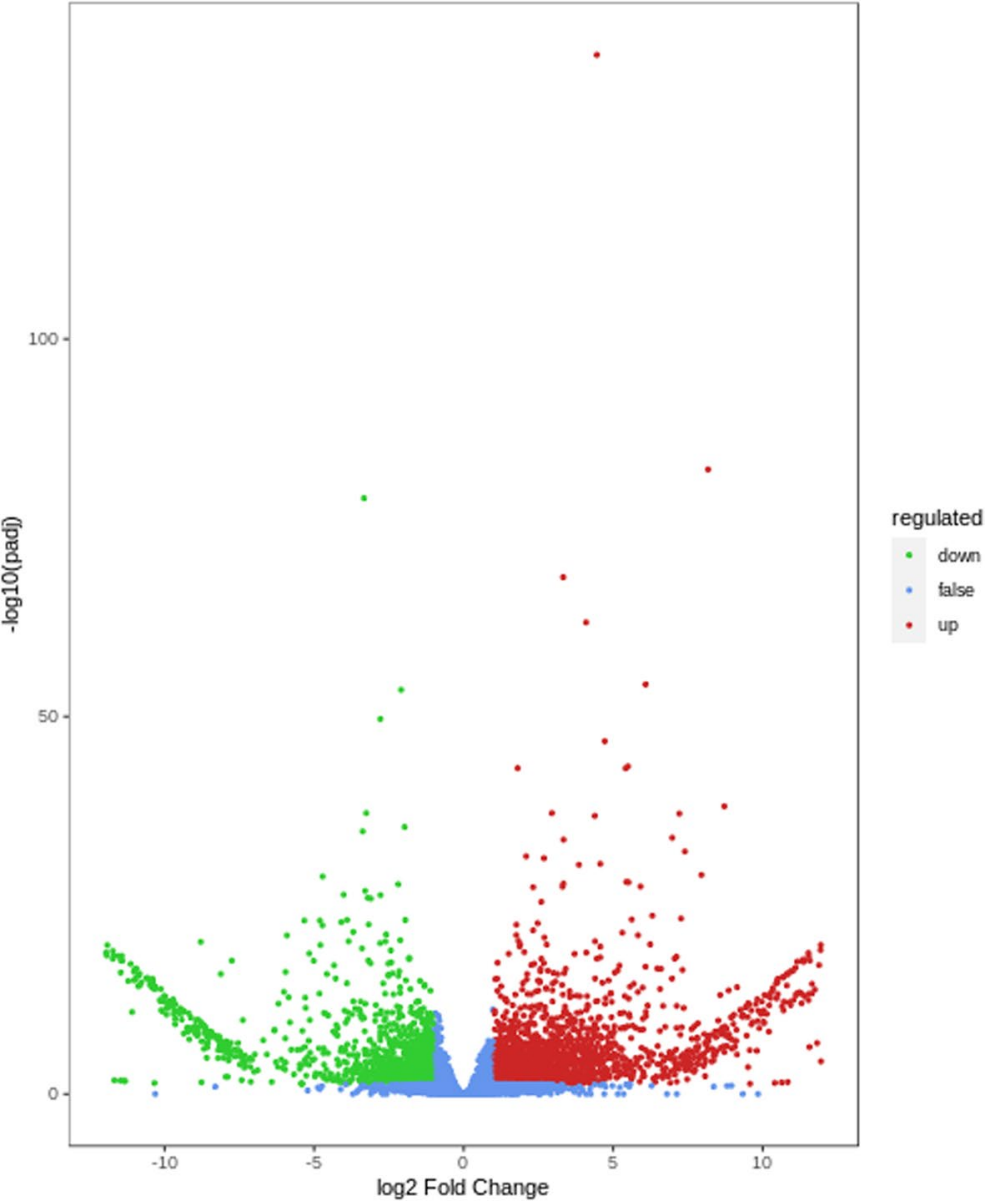
Volcano plot of differentially expressed genes in eggplant with green flesh and white flesh. Each point represents a DEG. In the plots, log_2_-transformed gene expression level ratios were plotted against the log_10_-transformed false discovery rate (FDR). Genes with FDR < 0.05 and fold changes above 1 were considered to be upregulated genes, which are shown in red. Genes with FDR < 0.05 and fold changes below -1 were considered to be downregulated genes, which are shown in green. The genes in blue did not show differential expression

Principal component analysis (PCA) of the samples based on fragments per kilobase of exon model per million reads mapped (FPKM) clearly separated the two flesh colour sample types, which indicated that the quality of the generated transcriptome data was high (Fig. 2A). Hierarchical cluster analysis was carried out on all the DEGs in the two eggplant varieties. The results showed that the expression patterns of most DEGs in NC7 and BL were completely opposite. Most of the genes expressed at higher levels in NC7 were expressed at lower levels in BL and vice versa (Fig. 2B). Of the DEGs obtained, 2050 were higher and 1254 were lower in BL (Fig. 3). Among the DEGs, 250 genes encoding transcription factors were identified. These transcription factors could be divided into 58 different common families. Of these families, bHLH (21), WRKY (17), NAC (17) and AP2-ERF (16) families involve a large number of TFs.

### Enrichment analysis of DEGs

**Fig. 4:**
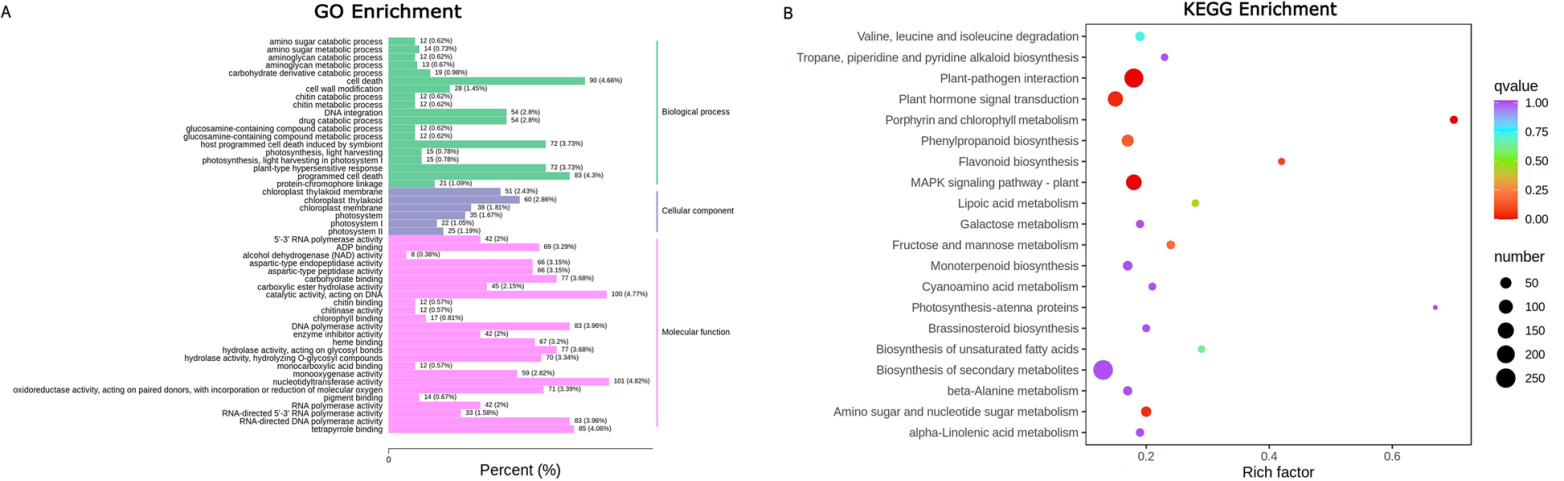
Gene functional enrichment analysis of DEGs in eggplant with green flesh and white flesh. **A** Gene Ontology (GO) enrichment analysis. The horizontal axis represents the ratio of genes annotated to the terms to the total number of genes annotated. The ordinate represents the name of the GO term. **B** Kyoto Encyclopedia of Genes and Genomes (KEGG) enrichment analysis. Each point in this chart indicates one KEGG pathway. The size of the point represents the number of DEGs enriched in the KEGG pathway. The colour of the point indicates the q-value, which is the *P* value after correction by the multiple hypothesis test; the smaller the q-value is, the more significant the KEGG pathway. Rich factor is the ratio of the proportion of DEGs annotated to a pathway among all DEGs to the proportion of genes annotated to that pathway among all genes

Gene Ontology (GO) enrichment analysis was performed for 3304 DEGs in the samples. Annotated DEGs were divided into fifty-four subclasses according to molecular function, biological process and cellular component. There were 10 subclasses under molecular function, 26 subclasses under biological process, and 18 subclasses under cellular component (Fig. 4A). It is worth noting that Unigenes involving chloroplasts and plastids accounted for the largest proportion in the cellular component. Kyoto Encyclopedia of Genes and Genomes (KEGG) annotation results showed that DEGs were assigned to 126 pathways, with porphyrin and chlorophyll metabolism, flavonoid biosynthesis, photosynthesis-antenna proteins playing vital roles in the colour formation of eggplant flesh (Fig. 4B).

### DEGs Involved in chlorophyll metabolism and photosynthesis – antenna proteins

**Fig. 5:**
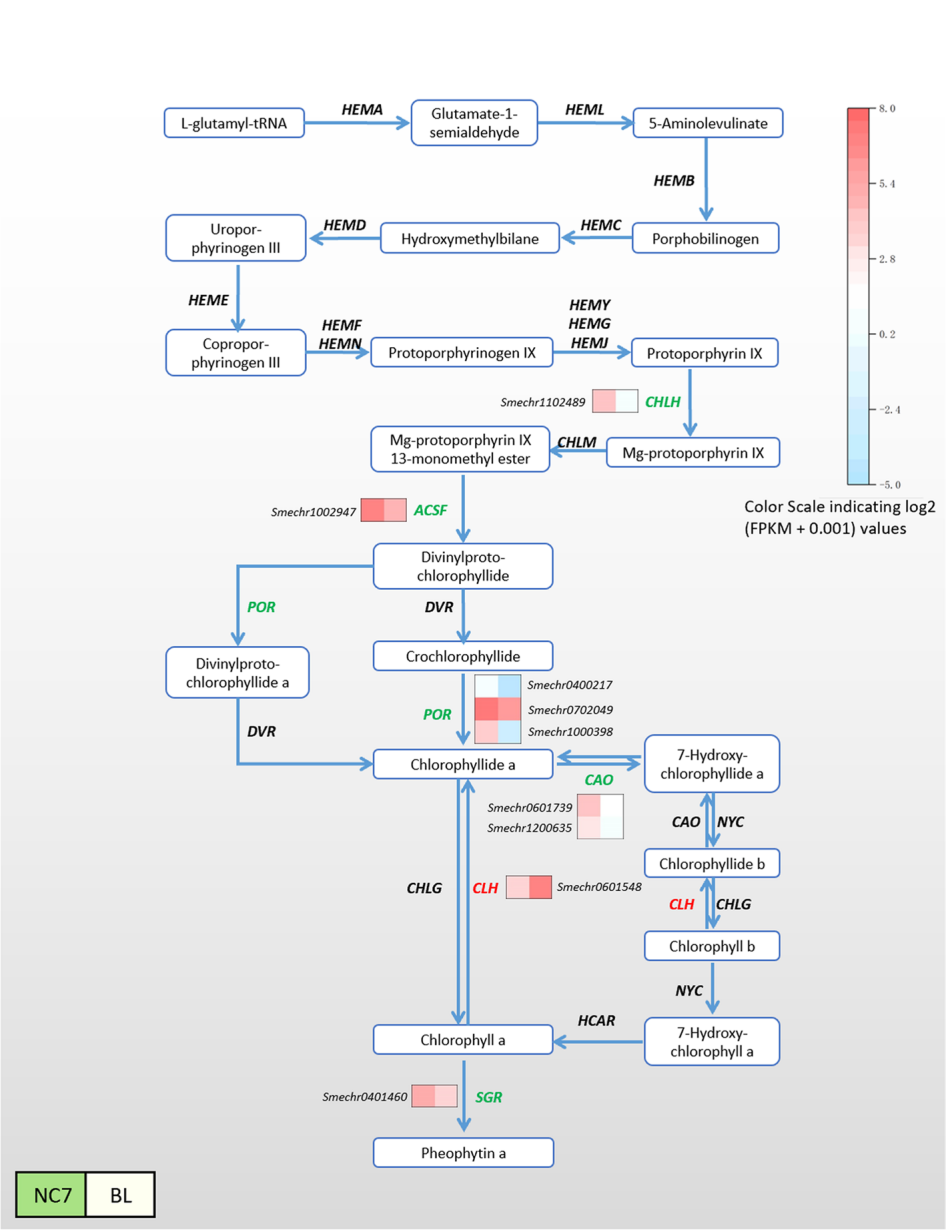
Expression of DEGs in the chlorophyll metabolism pathway. The heatmap columns from left to right are NC7 and BL. The colour scale indicates log_2_ (FPKM + 0.001) values. The colour change in this part only represents the DEGs expression level in each line of each line, and there was no comparability between different lines. *CHLH*: magnesium chelatase subunit H; *ACSF*: magnesium-protoporphyrin IX monomethyl ester cyclase; *POR*: protochlorophyllide reductase; *CAO*: chlorophyllide a oxygenase; *CLH*: chlorophyllase; *SGR*: Mg dechelatase. NC7: green-fleshed eggplant; BL: white-fleshed eggplant

A total of 9 homologous DEGs were related to the chlorophyll metabolism pathway, 89.9% of which were lower in BL (Fig. 5). In this pathway, compared with NC7, the expression levels of *CHLH, ACSF, POR, CAO* and *SGR* homologues were lower in BL. Meanwhile, only *CLH* homologues were higher. Among these, the expression levels of *POR* homologues were significantly lower (more than 40-fold).

**Fig. 6:**
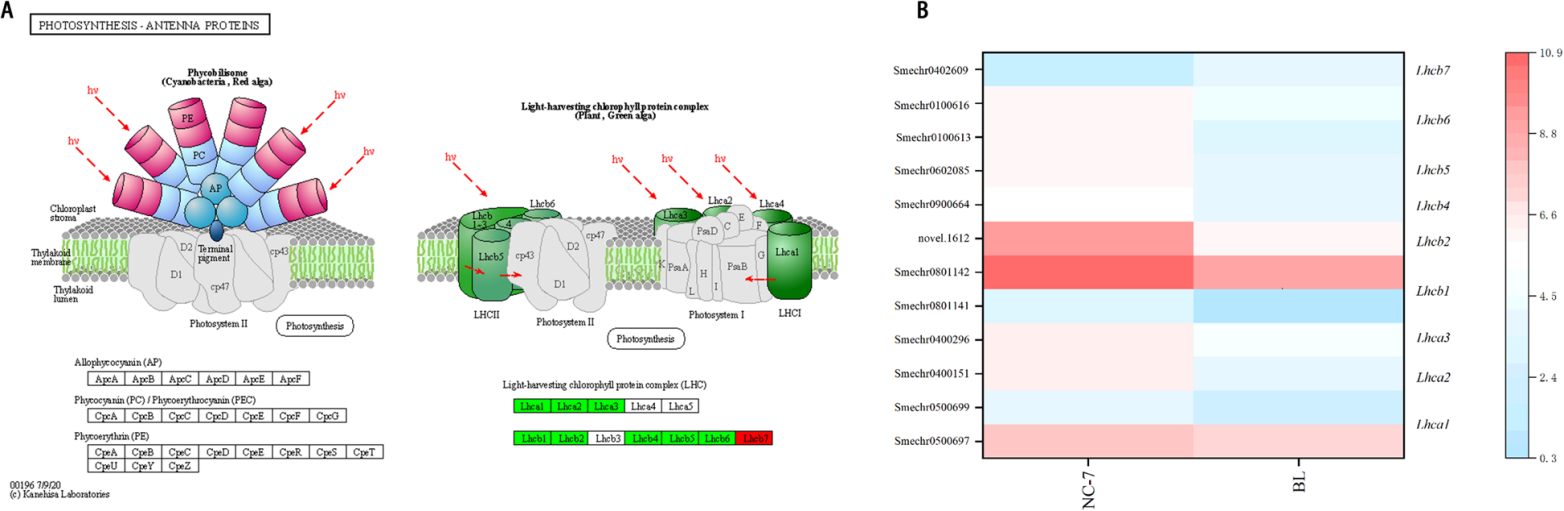
Expression of DEGs in photosynthesis – antenna proteins. **A** The photosynthesis – antenna protein pathway(This image is obtained by KEGG). **B** The expression levels of DEGs participating in the photosynthesis – antenna protein pathway. The colour scale indicates log_2_ (FPKM + 0.001) values. *Lhca1-3*: light-harvesting complex I chlorophyll a/b binding protein 1–3. *Lhcb1, 2, 4–7*: light-harvesting complex II chlorophyll a/b binding protein 1, 2, 4–7. NC7: green-fleshed eggplant; BL: white-fleshed eggplant

A total of 12 homologous DEGs were related to the photosynthesis-antenna protein pathway, 91.6% of which were lower in BL (Fig. 6A). In this pathway, compared with NC7, the expression levels of *Lhca1, Lhca2, Lhca3, Lhcb1, Lhcb2, Lhcb4, Lhcb5* and *Lhcb6* homologues were lower. Meanwhile, only *Lhcb7* homologues were higher (Fig. 6B). Overall, most of the DEGs related to chlorophyll and photosynthetic antenna protein synthesis were lower in eggplants with white flesh(BL). KEGG enrichment pathway analysis revealed that genes associated with chlorophyll and photosynthesis-antenna protein anabolism all showed repressed or decreased expression profiles. However, DEGs that promote chlorophyll and photosynthesis-antenna protein catabolism all exhibited abundant or increased expression.

### RNA-seq data validation

Based on the results of KEGG analysis in DEGs, 20 candidate genes involved in the chlorophyll metabolism and photosynthesis-antenna protein pathways were selected, and their expression levels based on the flesh colour of NC7 (green) and BL (white) were analysed using qRT–PCR. The strong correlation between the qRT–PCR results and the RNA-seq data (R^2^ = 0.9279, Figure S1) confirmed that the RNA-seq data obtained in this experiment were highly reliable.

**Fig. 7:**
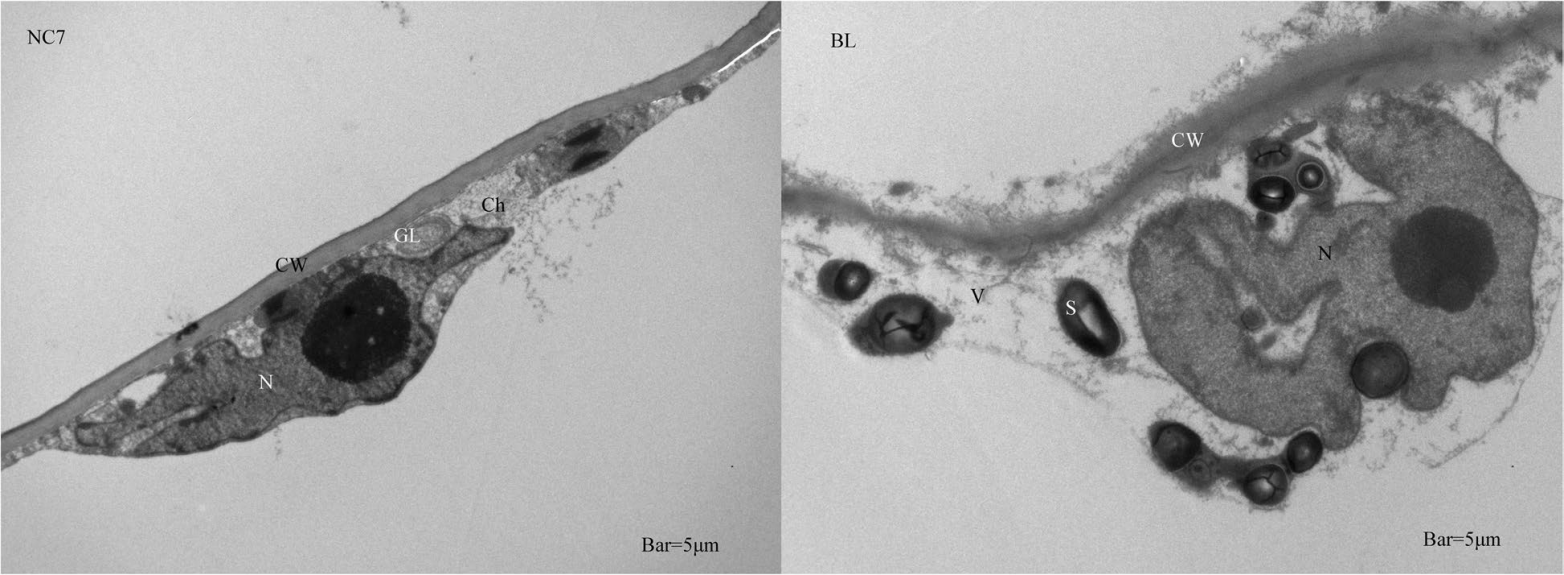
Comparison of cell structures in eggplant with different flesh colours. N: nucleus; CW: cell wall; GL: basal lamellae; Ch: chloroplast; V: vacuole; S: starch granules

**Fig. 8:**
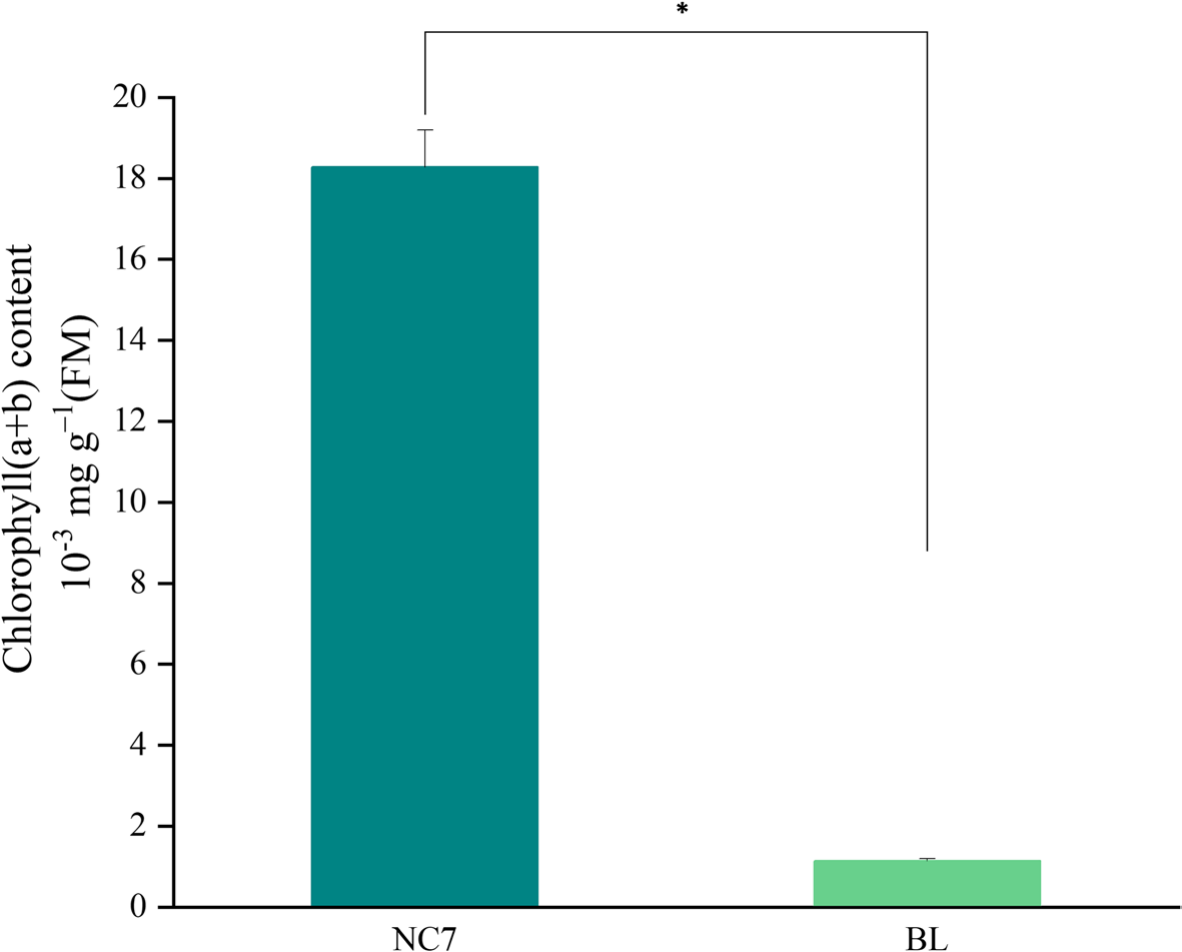
Chlorophyll contents in NC7 and BL. NC7: green-fleshed eggplant; BL: white-fleshed eggplant

A phenomenon worth noting in the GO enrichment results was that most DEGs related to chloroplast structure formation, such as chloroplast membrane and thylakoid, had lower expression level in BL. Therefore, the ultrastructure of flesh cells of NC7 and BL were observed to verify the reliability of the RNA-seq data in this study. As presented in Fig. 7, obvious basal lamellae (GL) were observed in NC7. In addition, chloroplasts (Ch) were found to adhere to cell walls (CW) during growth. Compared with NC7, no obvious chloroplasts were found in the flesh cells of all BL samples, and only leucoplasts with no obvious matrix thylakoids were observed. The determination of chlorophyll content in the flesh of NC7 and BL samples also confirmed these results (Fig. 8).

## Discussion

Previous reports have shown that anthocyanins determine the colouration of eggplant peel colour [4]. However, the flesh colour formation mechanism of eggplant has rarely been studied. The data from this study showed that the gap in chlorophyll content was the main source of the difference between white and green flesh in eggplant. Figure 8 shows that the chlorophyll content in NC7 (green) flesh was 16 times that of BL (white) flesh. Chlorophyll metabolism involves a series of enzymes, and changes in the expression levels of genes encoding these enzymes will directly affect the content of chlorophyll. The KEGG enrichment results in this study indicated that DEGs in the chlorophyll metabolic pathway were all located downstream of protoporphyrin IX. Except for *CLH*, the other five homologous genes expression level were all lower in BL. *CLH* plays a significant role as a rate-limiting enzyme in chlorophyll breakdown and regulates the conversion of chlorophyll a/b to chlorophyllide a/b. POR had lower expression level in BL, and a previous study found that downregulated expression of POR led to a decrease in the accumulation of chlorophyll a [32]. Mediated by the POR enzyme, prochlorophyll a undergoes a series of esterification and modification reactions to generate chlorophyll a and chlorophyll b [9]. *Porb-1-porc-1* double-mutant *A. thaliana* exhibited unstacked thylakoid membranes in its chloroplasts, indicating the yellow phenotype of seedling death. When the POR gene encoding PORA was overexpressed, it was confirmed that the double mutant *A. thaliana* could restore the *Porb-1-porc-1* phenotype and accumulate chlorophyll [33]. This means that the biosynthesis rate of chlorophyll was low and the degradation rate was high in BL (white) compared to NC7 (green), resulting in a white flesh colour. This was consistent with the process of lily petals turning from green to white [34]. In addition to the chlorophyll synthesis pathway, DEGs were also abundantly enriched in photosynthetic antenna proteins and the metabolic flavonoid pathway. The transcriptional results showed that the DEGs had lower expression level in BL involved in the photosynthesis-antenna protein pathway mainly included *LHCa* and *LHCb*. *LHCa* and *LHCb* (light-harvesting complex chlorophyll a/b binding protein) are the main components of the antenna system of higher plants. In particular, *LHCb* on the thylakoid membrane plays an important role in resisting protease degradation. The results of this study showed that most of the genes expression level related to photosynthetic antenna protein synthesis were lower in white-fleshed eggplant, suggesting that the blocked synthesis of light-harvesting tissues was directly linked to defective chlorophyll synthesis. This was verified in rice and tea [35, 36].

The chloroplast is the organelle where chlorophyll is synthesized. Abnormal chloroplast structure may be the cause of blocked chlorophyll synthesis or could be the result of the reduced demand for chloroplasts when chlorophyll is converted into other substances during fruit ripening. Through GO enrichment results, it was found that chloroplast membrane, thylakoid and thylakoid membrane were the three most abundant terms related to cell components. In addition, the number of decline expression level genes in the three terms far exceeded that of rise genes, indicating that the chloroplast tissue structure of BL (white-fleshed eggplant) was abnormal. In addition to the transcriptome data, the ultrastructure of eggplant with two flesh colours also confirmed this conclusion (Fig. 7). TEM results showed that only leucoplasts but no chloroplasts were found in the pulp cells of BL. Due to the distinct differentiation processes, the membrane structure inside the protoplasts in the pulp cells of BL did not form thylakoids but instead formed lamellar bodies, which only have storage function and do not have the ability to capture light. However, mature chloroplasts in dark environments may also transform into leucoplasts. Studies have shown that damage to thylakoid and lamellar structures in leaf chloroplast structure was the cause of the formation of albino tea leaves [37]. In this study, the pulp of BL eggplant appeared white throughout the growth period, indicating that the leucoplasts in the cells were caused by the failure of the differentiation of protoplasts into chloroplasts rather than the transformation of chloroplasts due to environmental conditions.

In this study, the pigment that made eggplant flesh green was mainly chlorophyll, and the metabolism of chlorophyll was often related to the mechanism of plant senescence [38–40]. The members of the ERF and bHLH transcription factor families are heavily involved in this regulatory process [41, 42]. PIF, a member of the bHLH family, is involved in activating chlorophyll degradation and inhibiting chloroplast activity under senescence and dark conditions in Arabidopsis [43]. Multiple bHLH family transcription factors were differentially expressed between early and late tepals of lily and had significant effects on changes in the chlorophyll content of tepals [41]. In research on chrysanthemum, bHLH family members were involved in regulating the metabolism of flavonoids and anthocyanins [44, 45], but there has been no report on their effect on the chlorophyll content in eggplant flesh. ERF is a family of plant-specific transcription factors that are widely involved in the regulation of plant growth [46], fruit ripening [47], and signal transduction [48] and in response to a variety of abiotic stresses [49]. Some studies have shown that ERF expression is negatively correlated with chlorophyll accumulation in fruits and leaves. In citrus fruits, CitERF6 and CitERF13 respond to ethylene signals, which in turn lead to chlorophyll degradation and fruit chlorosis [42]. Most of the genes related to chlorophyll synthesis were downregulated in Chinese cabbage etiolation leaves, while some transcription factors of the ERF family were upregulated [50]. Through transcription factor analysis, a total of 58 different transcription factor families were identified in eggplants with green and white flesh colours in this study, among which bHLH and ERF families are great contributors. These findings suggested that bHLH and ERF family members have important functions in eggplant pulp chlorophyll metabolism.

Another interesting finding was that, according to the data of another set of related experiments (unpublished data), the content of chlorogenic acid in NC7 (green) was 7.4 times higher than that in BL (white). Chlorogenic acid has a positive impact on human health [3]. Does this mean that NC7 has the potential to be developed as a health-care eggplant? We will focus on this possibility in future research.

## Conclusion

By comparing the transcriptome data of green- and white-fleshed eggplants, we found that differences in gene expression of the chlorophyll metabolic pathway were the main cause of the different flesh colour formations. Meanwhile, antenna protein synthesis and photosynthetic metabolism pathways also had important effects on the colour composition of eggplant flesh. In addition, chloroplast synthesis was hindered in the cytoplasm of white-fleshed eggplant. Several transcription factors, mainly ERF and bHLH family members, were predicted to regulate chlorophyll biosynthetic genes. These findings could provide a theoretical basis for eggplant variety directional improvement and explore the mechanism of fruit ripening.

## Supplementary Information

**Additional file 1: Figure S1.** qRT-PCR results of the selected genes and correlation between transcriptome data and real time PCR results.

**Additional file 2: Table S1.** Data qualities statistics of RNA-seq in eggplant flesh.

**Additional file 3: Table S2.** DEGs between NC7 and BL.

**Additional file 4: Table S3.** Primers used in the q-PCR analysis.

## Data Availability

Extra data has been appended as supplementary Tables and Figures. The accession number for sequence data generated in this study is PRJNA851536 available at https://www.ncbi.nlm.nih.gov/bioproject/PRJNA851536.

## Declarations

All the plant materials in this paper comply with relevant institutional, national, and international guidelines and legislation.
