# Supplementary material for: Transcriptomics reveals the molecular mechanisms of flesh colour differences in eggplant (Solanum melongena)
Source: BMC Plant Biol. 2023 Jan 4;23:5. doi: 10.1186/s12870-022-04002-z (PMC9811765; doi:10.1186/s12870-022-04002-z)
Supplement: Supplementary file 1 — Additional file 1: Figure S1. qRT-PCR results of the selected genes and correlation between transcriptome data and real time PCR results. [file 12870_2022_4002_MOESM1_ESM.docx]

**Figure S1** qRT-PCR results of the selected genes and correlation between transcriptome data and real time PCR results.
